# MAPK inhibitors induce serine peptidase inhibitor Kazal type 1 (SPINK1) secretion in BRAF V600E‐mutant colorectal adenocarcinoma

**DOI:** 10.1002/1878-0261.12160

**Published:** 2017-12-27

**Authors:** Kati Räsänen, Kien X. Dang, Harri Mustonen, Tho H. Ho, Susanna Lintula, Hannu Koistinen, Ulf‐Håkan Stenman, Caj Haglund, Jakob Stenman

**Affiliations:** ^1^ Department of Clinical Chemistry Medicum Helsinki University Hospital University of Helsinki Finland; ^2^ Minerva Foundation Institute for Medical Research Helsinki Finland; ^3^ Department of Surgery Helsinki University Hospital University of Helsinki Finland; ^4^ Department of Genomics BPARC Vietnam Military Medical University Hanoi Vietnam; ^5^ Research Program Unit Translational Cancer Biology University of Helsinki Finland; ^6^ Department of Pathology Helsinki University Hospital University of Helsinki Finland; ^7^ Department of Women's and Children's Health Karolinska Institutet Stockholm Sweden

**Keywords:** biomarker, BRAF V600E, colorectal cancer, inhibitor, SPINK1, trametinib

## Abstract

The mitogen‐activated protein kinase (MAPK) pathway plays a central role in colorectal cancers (CRC). In particular, BRAF V600E‐mutant tumors, which represent around 10% of CRCs, are refractory to current therapies. Overexpression and secretion of serine peptidase inhibitor Kazal type 1 (SPINK1) are observed in around 50% of CRCs, and its serum level can be used as a biomarker for poor prognosis. Utilizing a recently developed extendable blocking probe assay, we analyzed the BRAF mutation status in a CRC patient cohort (*N* = 571) using tissue‐derived RNA as the starting material. From the same RNA samples, we measured the relative SPINK1 expression levels using a quantitative real‐time PCR method. Expression of mutant BRAF V600E correlated with poor prognosis, as did low expression of SPINK1 mRNA. Further, BRAF V600E correlated negatively with SPINK1 levels. In order to investigate the effect of MAPK pathway‐targeted therapies on SPINK1 secretion, we conducted *in vitro* studies using both wild‐type and V600E CRC cell lines. BRAF inhibitor vemurafenib, and subsequent MAPK pathway inhibitors trametinib and SCH772984, significantly increased SPINK1 secretion in V600E CRC cell lines Colo205 and HT‐29 with a concomitant decrease in trypsin‐1 and ‐2 secretion. Notably, no SPINK1 increase or trypsin‐1 decrease was observed in BRAF wild‐type CRC cell line Caco‐2 in response to MAPK pathway inhibitors. In further mechanistic studies, we observed that only trametinib was able to diminish completely both MEK and ERK phosphorylation in the V600E CRC cells. Furthermore, the key regulator of integrated stress response, activating transcription factor 4 (ATF‐4), was downregulated both at mRNA and at protein level in response to trametinib treatment. In conclusion, these data suggest that sustained inhibition of not only MAPK pathway activation, but also ATF‐4 and trypsin, might be beneficial in the therapy of BRAF V600E‐mutant CRC and that SPINK1 levels may serve as an indicator of therapy response.

**Abbreviations**
ADAM: a disintegrin and metalloproteinases
ATF‐4: activating transcription factor 4
CRC: colorectal cancer
EGFR: epidermal growth factor receptor
ExBP‐RT: extendable blocking probe reverse transcriptase
FFPE: formalin‐fixed paraffin‐embedded
IFMA: immunofluorometric assay
IHC: immunohistochemistry
IQR: interquartile range
MAPK: mitogen‐activated protein kinase
qPCR: quantitative PCR
SPINK1: serine peptidase inhibitor Kazal type 1
TIMP1: tissue inhibitor of metalloproteinases 1

## 1 Introduction

Colorectal cancer (CRC) is the third most common cancer in men and the second in women worldwide. While about 20% of patients have disseminated disease at diagnosis, part of the patients initially classified as having regional disease and even some of those with local disease will develop a recurrence and eventually die from cancer. Overall 5‐year survival is 50–60% (Siegel *et al*., 2012). Current FDA‐approved molecularly targeted therapies for metastasized CRC include several monoclonal antibodies against vascular endothelial growth factor and epidermal growth factor receptor (EGFR), a multikinase small‐molecule inhibitor regorafenib, and a nucleoside analog trifluridine/tipiracil (Moriarity *et al*., 2016). Currently available EGFR antibodies cetuximab and panitumumab bind to the extracellular domain of EGFR resulting in receptor internalization and blockage of signaling. Mutations in the RAS family of proto‐oncogenes (KRAS, NRAS, HRAS) result in constitutive activation of the mitogen‐activated protein kinase (MAPK) pathway signaling independent of activation of receptor tyrosine kinases such as EGFR. Therefore, mutations in KRAS or NRAS cause intrinsic resistance to EGFR‐targeted therapies (Semrad and Kim, 2016).

V600E mutation in BRAF, which is immediately downstream of RAS, has also been proposed to cause resistance to anti‐EGFR antibodies. This mutation is found in about 10% of CRCs leading to an aggressive subtype, for which there is no effective oncological therapy (Dienstmann and Tabernero, 2016). Unexpectedly, specific BRAF V600E inhibitors, such as vemurafenib that is highly effective in melanoma, do not benefit patients with CRC. Subsequently, it was shown that intrinsic resistance to vemurafenib in CRC is caused by EGFR autoactivation through an unknown mechanism (Prahallad *et al*., 2012). In recent clinical trials, a combination of a BRAF V600E inhibitor with a MEK (Corcoran *et al*., 2015) or a PI3K (Elez *et al*., 2015) inhibitor showed a clinical benefit. Therefore, sustained MAPK inhibition appears to be a critical determinant of the clinical benefit, and differing from melanoma, it seems that only combinations are able to generate therapeutic effects in CRC (Dienstmann and Tabernero, 2016).

Overexpression and secretion of serine peptidase inhibitor Kazal type 1 (SPINK1, also known as tumor‐associated trypsin inhibitor, or pancreatic secretory trypsin inhibitor) are observed in a variety of cancers (Räsänen *et al*., 2016a). In CRC, increased SPINK1 serum levels are found in around 50% of patients, and it is an independent prognostic factor (Gaber *et al*., 2010). However, the prognostic value of tissue expression of SPINK1 in CRC is controversial, as depending on study it has been predictive of either poor or good prognosis, or nonsignificant (Chen *et al*., 2015; Koskensalo *et al*., 2013 (Ida *et al*., 2015). We have previously shown that concomitant SPINK1 and EGFR expression in CRC tissue predicts favorable prognosis (Koskensalo *et al*., 2013); Koskensalo *et al*. (2012) and Chen *et al*. (2016) showed that high tissue expression of SPINK1 in CRC correlated with a better prognosis.

In addition to intrinsic resistance, acquired resistance presents a clinical problem as a majority of the patients who are treated with molecularly targeted treatments relapse within a year. Therefore, both novel methods and novel biomarkers that predict treatment response are needed for the stratification of patients in order to select appropriate therapy. In the current study, we investigated the expression of BRAF V600E mutations in a CRC cohort of 571 patients using a novel extendable blocking probe reverse transcriptase (ExBP‐RT) assay that we recently developed. ExBP‐RT is an ultra‐high selective method and allows for analysis of expressed mutations at the RNA level (Ho *et al*., 2015). This method therefore enables analysis of mutational status irrespective of whether the mutation is inherited or acquired. The use of tumor tissue RNA instead of DNA as the starting material enabled us to correlate the mRNA expression of BRAF V600E mutations directly with SPINK1 mRNA expression level analyzed by qPCR from the same tumor samples. Further, using BRAF wild‐type and V600E CRC cell lines, we studied the effects of MAPK inhibitors on SPINK1 secretion *in vitro*.

## 2 Materials and methods

### 2.1 RNA samples

RNA was extracted as described (Ho *et al*., 2015) from formalin‐fixed, paraffin‐embedded (FFPE) samples from patients who were operated for histologically confirmed CRC at the Department of Surgery, Meilahti Hospital, Helsinki University Hospital between 1987 and 2003. In total, 571 patients’ samples were available for this study. The use of clinical samples for this purpose was approved by the Surgical Ethics Committee of Helsinki University Hospital and the National Supervisory Authority of Welfare and Health and collected from the archives of the Department of Pathology, Helsinki University Hospital. All RNA samples were quantified with a NanoVue spectrophotometer (GE Healthcare, Waukesha, WI, USA). Table 1 describes the clinicopathological features of the cohort.

**Table 1:** Patient characteristics

All patients	*N* = 571	*n*	%
Age	< 65	231	40
	≥ 65	340	60
Gender	Female	264	46
	Male	307	54
Type	Adeno	513	90
	Mucinous	58	10
Location	Colon	381	67
	Rectum	190	33
Side	Dex	201	35
	Sin	369	65
Dukes	A	74	13
	B	211	37
	C	166	29
	D	120	21
Grade	1	28	5
	2	392	69
	3	109	19
	4	21	4
Age (min–max), years	68.1	(29.3–97.2)	

### 2.2 ExBP‐RT assay

The extendable blocking probe method (Ho *et al*., 2015) was employed for ultrasensitive detection of the BRAF V600E gene mutation. Using RNA templates, this novel method allows for the detection of expressed mutations in at least a 1000 times higher background of the corresponding wild‐type alleles. The principles of ExBP‐RT assays and reaction setup procedures for multiplex detection of BRAF V600E mutation were as described in the original paper (Ho *et al*., 2015).

RNA extracted from FFPE samples was diluted to 100 ng·μL^−1^ in DEPC H_2_O for the ExBP‐RT assay, before the allele‐specific reverse transcription reaction. RNA extracted from Colo205 (BRAF V600E mutant) and A549 (BRAF wild‐type) cell lines were used as positive and negative controls, respectively, in ExBP‐RT assays of BRAF mutation detection. All control RNAs were extracted from cultured cells using RNA/DNA purification Kit (Norgen Biotek, Thorold, ON, Canada), quantified with a NanoVue spectrophotometer (GE Healthcare), and diluted to 100 ng·μL^−1^ in DEPC H_2_O.

Using cDNA products of the ExBP‐RT assays as a template, the real‐time PCR amplification was performed to detect/quantify the expression of mutant BRAF V600E. QuantiTect Probe PCR Kits (Qiagen, Hilden, Germany) were used for these probe‐based real‐time PCR assays according to the manufacturer's instructions in a 10 μL reaction volume. A common reverse primer was designed to target the 5′‐prime tail of all mutation‐specific ExBP‐RT products. The expression levels of total BRAF genes (including V600E mutant and its wild‐type segments) were also determined in each sample for normalization using QuantiTect SYBR Green PCR Kits (Qiagen) according to the manufacturer's instructions in a 10 μL volume. The sequences and concentration of qPCR primers and probes are provided in Table 2. The same thermocycling conditions were used for both probe‐based and SYBR Green‐based real‐time quantitative PCR (qPCR): 95 °C for 15 min, 45 cycles at 94 °C for 10 s, at 60 °C for 45 s. Following SYBR Green‐based qPCR, the specificity of the amplification products was verified by melting curve analysis. All qPCR assays were run on a LightCycler 480 II Real‐Time PCR Instrument (Roche Applied Science, Mannheim, Germany) with a 384‐well white‐plate (Roche Applied Science). All mutation, wild‐type, and H_2_O controls of each experiment were checked to verify the results in both ExBP‐RT and qPCR assays. Threshold cycle (*C*_t_) values of qPCR were calculated automatically using the absolute quantification analysis with the fit points method, which is built in the LightCycler 480 II system. The method allows to setting the noise band and the threshold line in order to discard uninformative background noise.

**Table 2:** Primer and probe sequences for qPCR step of different ExBP‐RT assays (locked nucleic acid (LNA) = [+A], [+G], [+C], [+T]; inosine = i; 6‐carboxyfluorescein: FAM; black hole quenchers: BHQ)

Primers and probes	Sequences (5′–3′)	Concentrations, μm
Mutant BRAF V600E assays		
BRAF forward primer	5′‐AGACCTCACAGTAAAAATAGGTGA‐3′	0.5
Common reverse primer	5′‐CGATCAGACGACGAC‐3′	0.5
BRAF‐Probe	FAM‐TTC[+T]CT[+G]TA[+G]CT[+A]GACCAA‐BHQ1	0.1
Total BRAF assays		
Total BRAF forward primer	5′‐CATGAAGACCTCACAGTAAA‐3′	1.5
Total BRAF reverse primer	5′‐GATTTCACTGTAGCTAGACC‐3′	1.5

### 2.3 Real‐time quantitative PCR

For FFPE samples, 500 ng of total RNA was reverse‐transcribed with 100 U Revert Aid Premium Reverse Transcriptase (Thermo Fisher Scientific, Waltham, MA, USA) using 4 pmol of gene‐specific antisense primers for *SPINK1* and *RPL13A* (see below for sequences), 0.5 mm dNTP mix, and 20 U Ribolock RNAse inhibitor (all from Thermo Fisher Scientific). Possible contamination of RNA in FFPE‐extracted samples with SPINK1 or RPL13A DNA was excluded by subjecting each sample to RT reaction without Revert Aid Premium Reverse Transcriptase. Real‐time qPCR was performed with a LightCycler 480 II instrument using a 384‐well thermal block (Roche Applied Science) with SensiFAST SYBR No‐ROX Kit (Bioline, London, UK). *SPINK1*,* PRSS1,* and *PRSS2* qPCR from cell lines was performed using the conditions described previously (Räsänen *et al*., 2016b). The following primers, purchased from TAG Copenhagen (Copenhagen, Denmark) and verified earlier (Räsänen *et al*., 2016b), were used: *SPINK1* forward 5′‐TGT CTG TGG GAC TGA TGG AA, *SPINK1* reverse 5′‐GCC CAG ATT TTT GAA TGA GG, *PRSS1* forward 5′‐CCA CCC CCA ATA CGA CAG GAA G, *PRSS1* reverse 5′‐GCG CCA GAG CTC GCA GT, *PRSS2* forward 5′‐CCA AAT ACA ACA GCC GG, *PRSS2* reverse 5′‐AGT CGG CAC CAG AAC TCA GA, *RPL13A* forward 5′‐AGA TGG CGG AGG TGC AG and *RPL13A* reverse 5′‐GGC CCA GCA GTA CCT GTT TA.

Following SYBR Green‐based qPCR, the specificity of the amplification products was verified by melting curve analysis and a control sample was included in every run to confirm interassay reproducibility. All reactions were run in duplicate, and for all samples, RT‐controls were run to exclude possible DNA contamination. Relative expression of target gene mRNA referenced to RPL13A housekeeping gene was calculated using the ∆∆*C*_t_ method (Livak and Schmittgen, 2001).

Human transcription factors (non‐HOX) TaqMan Array (Thermo Fisher Scientific) that contains 92 assays for non‐HOX transcription factor‐associated genes and four assays for endogenous control genes were performed according to the manufacturer's protocol.

### 2.4 Immunohistochemistry

SPINK1 immunohistochemistry (IHC) was performed as described recently (Räsänen *et al*., 2016b). Briefly, FFPE sections were deparaffinized with xylene and rehydrated in ethanol series. Mouse monoclonal anti‐SPINK1 (2 μg·mL^−1^, in‐house clone 6E8) (Osman *et al*., 1993) was incubated overnight at 4 °C. Isotype control antibody was mouse IgG #I‐2000 (Vector Laboratories, Burlingame, CA, USA). Mach 4 Universal AP‐Polymer kit (Biocare Medical, Concord, CA, USA) was used for detection, and the visualization signal was developed with Vector Red Alkaline Phosphatase Substrate kit (Vector Laboratories).

### 2.5 Cell culture

BRAF V600E CRC cell lines Colo205 and HT‐29 (American Type Culture Collection, Manassas, VA, USA), and BRAF wild‐type cell lines Caco‐2, and SW‐480 (Sigma‐Aldrich, St. Louis, MO, USA), were cultured and authenticated as previously described (Räsänen *et al*., 2016b). Melanoma cell lines SK‐MEL‐2 (BRAF wild‐type) and SK‐MEL‐5 (BRAF V600E) were purchased from American Type Culture Collection and grown in EMEM. All cell lines were cultured at +37 °C in 5% CO_2_ atmosphere and supplemented with 5% fetal bovine serum (Biowest, Nuaille, France), 0.3 mg·mL^−1^ glutamine, 100 μg·mL^−1^ streptomycin, and 100 U·mL^−1^ penicillin (all from Lonza, Basel, Switzerland). Cells were used until passage number 20 and routinely tested for mycoplasma.

### 2.6 Inhibitors

The following inhibitors were purchased from Selleck Chemicals (Munich, Germany) and dissolved in DMSO according to the manufacturer's instructions: gefitinib (EGFR), GW5074 (CRAF), LY294002 (AKT), PD98059 (MEK1), salisarib (RAS), SCH772984 (ERK1/2), trametinib (MEK1/2), vemurafenib (BRAF), and zoledronic acid (RAS and RHO).

### 2.7 Immunofluorometric assay

Time‐resolved immunofluorometric assays (IFMA) developed in‐house for SPINK1, trypsinogen‐1, and trypsinogen‐2 (Itkonen *et al*., 1990; Janeiro *et al*., 2012; Koivunen *et al*., 1990; Paju *et al*., 2001) were performed as described previously (Räsänen *et al*., 2016b). The concentrations of secreted proteins were measured from 24‐, 48‐, and 72‐h conditioned media with or without various inhibitor treatments. The detection ranges for the IFMAs are the following: SPINK1 0.5–90 ng·mL^−1^, trypsinogen 1 1.6–400 ng·mL^−1^, and trypsinogen 2 2–500 ng·mL^−1^.

### 2.8 Western blotting

Samples for western blot analysis were harvested as described (Räsänen *et al*., 2008). Samples were run on 4–12% gradient gels (Life Technologies, Carlsbad, CA, USA). Using Trans‐Blot Turbo system, proteins were transferred to nitrocellulose membrane (both from Bio‐Rad, Hercules, CA, USA) and blocked with 5% (w/v) nonfat powdered milk in TBS (20 mm Tris/HCl pH 7.5, 150 mm NaCl, and 0.1% Tween‐20). Immunoreactive proteins were visualized with appropriate primary and secondary antibodies using ECL detection (Bio‐Rad).

The following primary antibodies were used according to the manufacturers’ recommended dilutions: rabbit monoclonal anti‐phospho‐p44/42 MAPK (ERK1/2, Thr202/Tyr204), rabbit polyclonal anti‐p44/42 MAPK (ERK1/2), rabbit monoclonal anti‐phospho‐MEK1/2 (Ser217/221), mouse monoclonal anti‐MEK1/2 (L38C12), rabbit polyclonal anti‐phospho‐STAT3 (Y705), mouse monoclonal anti‐STAT3 (124H6), and rabbit monoclonal anti‐ATF4 (D4B8) (all from Cell Signaling Technology, Danvers, MA, USA). Rabbit polyclonal anti‐GAPDH was from Sigma‐Aldrich. The secondary antibodies used in western blotting were affinity‐purified horseradish peroxidase‐coupled anti‐rabbit IgG H+L and anti‐mouse IgG H+L (both from Jackson ImmunoResearch, Suffolk, UK).

### 2.9 Statistical analysis

Results are given as the number of patients and percentage or mean and SD or median and range or interquartile range (IQR). The Fisher's exact test and the linear‐by‐linear association test were used to assess associations between clinicopathological variables and mutation status or mRNA expression. The relative SPINK1 mRNA expression was dichotomized at 87.5% percentile. Differences in continuous variables between different groups were tested with the unpaired *t*‐test or with the Mann–Whitney test or in the case of ordinal grouping variable with the Jonckheere–Terpstra test. Survival analysis was performed with the Kaplan–Meier method, and the log‐rank test was used to compare the groups. The Cox regression proportional hazard model was used for uni‐ and multivariate survival analyses. Multivariate analysis was adjusted for age, gender, stage, and location. Interaction terms were considered. The Cox model assumption of constant hazard ratios over time was tested. A time‐dependent covariate was included separately for each testable variable at a time. A time‐dependent correction factor was included in the models, if the hazard ratio was not constant over time. The Spearman's rho correlation coefficient was calculated to assess the correlation between continuous and ordinal variables. All *in vitro* experiments were conducted in duplicate and repeated three times. *P‐*values of less than 0.05 were considered to be statistically significant, and two‐tailed tests were used. Statistical analyses were carried out with spss (version 24; IBM, New York, NY, USA) and GraphPad software (La Jolla, CA, USA).

## 3 Results

### 3.1 BRAF V600E mutation detection by ExBP‐RT assay

**Figure 1:**
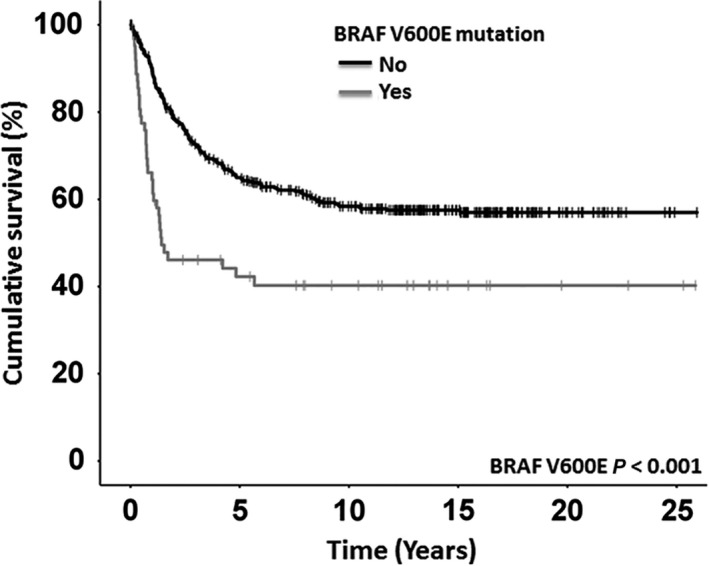
Survival curves for BRAF wild‐type‐ and V600E‐expressing colorectal cancer patients.

BRAF V600E mutation is known to be a strong marker of poor prognosis in metastatic CRC. The prevalence of this mutation is around 10% (Dienstmann and Tabernero, 2016). We detected expressed BRAF V600E mutations in 8% of the samples in our cohort, and the presence of BRAF V600E correlated significantly with poor prognosis (the log‐rank test, *P* < 0.001). Patients with no detected expression of V600E mutations had a mean survival time of 16.1 (95% CI, 15.0–17.2) years compared to 11.1 (8.0–14.2) years for patients with expressed mutations (Fig. 1). In univariate Cox analysis, the hazard ratio for expressed BRAF V600E mutations was 2.1 (95% CI, 1.5–3.0, *P* < 0.001), and in multivariate model after adjusting for age, gender, stage, and location, it was 2.8 (95% CI, 1.8–4.4, *P *= < 0.001) (Table 3).

**Table 3:** Statistical analyses of expressed BRAF V600E mutations and SPINK1 expression

	HR	95% CI		*P*
		Lower	Upper	
Univariate				
Age 65 year, age = > 65 vs < 65	1.45	1.11	1.90	0.006
Gender, male vs female	1.03	0.80	1.34	0.807
Dukes				
A	1.00			
B	2.09	0.98	4.45	0.056
C	6.52	3.15	13.49	0.000
D	27.92	13.48	57.84	0.000
Grade				
1	1.00			
2	1.96	0.87	4.42	0.106
3	2.88	1.24	6.71	0.014
4	3.09	1.12	8.50	0.029
Location, rectum vs colon	1.25	0.96	1.64	0.095
Side, sin vs dex	1.25	0.95	1.66	0.116
Type, mucinous vs adeno	0.93	0.61	1.42	0.744
BRAF V600E mutation, yes vs no	2.12	1.48	3.03	0.000
SPINK1 mRNA, continuous	0.93	0.85	1.01	0.098
SPINK1 mRNA dichotomous, > 2.4 vs ≤ 2.4	0.556	0.321	0.961	0.036
SPINK1 mRNA				
< 0.5	1			
0.5–2.4	0.87	0.63	1.20	0.403
≥ 2.4	0.51	0.29	0.91	0.023
BRAF V600E and SPINK1 mRNA				
BRAF V600E mutation	1.00			
SPINK1 ≤ 2.4, no mutation in BRAF	0.40	0.26	0.62	0.000
SPINK1 > 2.4, no mutation in BRAF	0.23	0.12	0.45	0.000
Multivariate BRAF				
Age 65 year, age = > 65 vs < 65	2.01	1.52	2.66	0.000
Gender, male vs female	1.14	0.87	1.48	0.344
Dukes				
A	1			
B	2.17	1.01	4.66	0.047
C	7.08	3.39	14.76	0.000
D	32.16	15.36	67.33	0.000
Location, rectum vs colon	1.47	1.12	1.93	0.006
BRAF V600E mutation, yes vs no	2.84	1.84	4.40	0.000
BRAF V600E time dependent, after 2 years of survival	0.16	0.05	0.54	0.003
Multivariate SPINK1				
Age 65 year, age = > 65 vs < 65	2.20	1.58	3.07	0.000
Gender, male vs female	1.12	0.81	1.54	0.489
Dukes				
A	1			
B	2.68	1.02	7.02	0.045
C	8.71	3.44	22.06	0.000
D	40.93	16.03	104.52	0.000
Location, rectum vs colon	1.66	1.19	2.32	0.003
SPINK1 mRNA				
< 0.5	1			
0.5–2.4	0.72	0.50	1.02	0.061
≥ 2.4	0.43	0.22	0.84	0.014
SPINK1 mRNA time dependent, after five years of survival	2.48	1.13	5.41	0.023
Multivariate SPINK1 and BRAF				
Age 65 year, age = > 65 vs < 65	2.12	1.51	2.96	0.000
Gender, male vs female	1.13	0.82	1.55	0.465
Dukes				
A	1			
B	2.77	1.06	7.28	0.038
C	8.79	3.47	22.26	0.000
D	43.59	17.02	111.65	0.000
Location, rectum vs colon	1.75	1.25	2.45	0.001
BRAF V600E and SPINK1 mRNA				
BRAF V600E mutation	1			
SPINK1 ≤ 2.4, no mutation in BRAF	0.29	0.17	0.47	0.000
SPINK1 > 2.4, no mutation in BRAF	0.09	0.03	0.27	0.000
BRAF V600E and SPINK1 mRNA, after 2 years of survival	3.49	1.43	8.54	0.006

### 3.2 Low expression of tumor SPINK1 mRNA associates with poor prognosis

**Figure 2:**
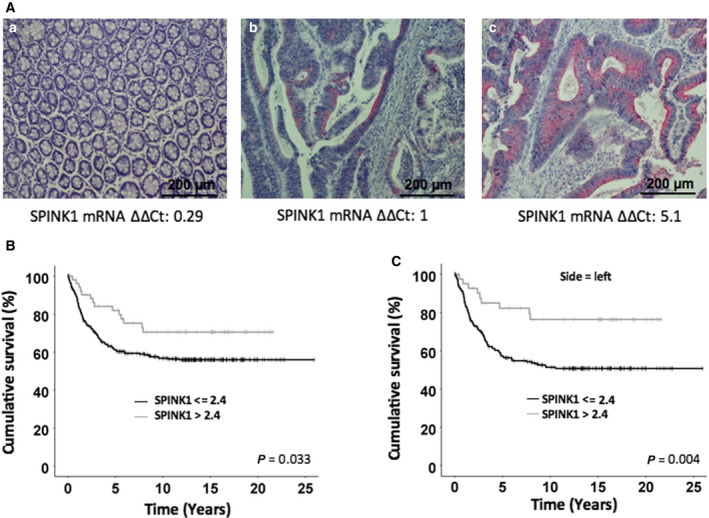
(A) Representative IHC images of SPINK1 (a) negative, (b) positive (< 2.4), and (c) positive (> 2.4) immunoexpression. Images taken at 10× magnification. The value below is the relative SPINK1 mRNA expression in the same sample calculated using the ∆∆*C*_t_ method. (B) Survival curves for SPINK1 mRNA (< 2.4) and (> 2.4) colorectal cancer patients. (C) Survival curves for SPINK1 mRNA (< 2.4) and (> 2.4) colorectal cancer patients having disease on the left side.

First, we correlated the SPINK1 mRNA qPCR data with the previously published (Koskensalo *et al*., 2012) immunohistochemistry result of this cohort. The relative expression level of SPINK1 measured by qPCR significantly correlated with the previous IHC results (Spearman's rho 0.366, *P* < 0.001, *n* = 242). Representative images of SPINK1 IHC and corresponding relative SPINK1 mRNA levels are shown in Fig. 2A. Further, in line with the reported result of the IHC staining where low SPINK1 immunoreactivity was an independent prognostic factor for adverse outcome (Koskensalo *et al*., 2012), low SPINK1 mRNA expression was associated with poor prognosis (Fig. 2B, the log‐rank test, *P* = 0.033). This was more prominent in patients having disease on the left side (Fig. 2C, the log‐rank test *P* = 0.004). Patients with high SPINK1 mRNA level (> 2.4 on a relative scale) and left‐side disease had a mean survival time of 17.4 (95% CI, 14.9–19.8) years compared to 14.5 (95% CI, 12.9–16.1) years for patients with lower SPINK1 mRNA level (≤ 2.4). In univariate Cox analysis, the hazard ratio for SPINK1 mRNA level was 0.51 (95% CI, 0.29–0.91, *P* = 0.023, relative mRNA expression = > 2.4 vs < 0.5) and 0.43 (95% CI, 0.22–0.84, *P* = 0.014) in multivariate model adjusted for age, gender, stage, and location (Table 3).

### 3.3 Correlation of expressed BRAF V600E mutations with SPINK1 expression

As the independent analyses of expressed BRAF V600E mutations and low SPINK1 expression were indicative of poor prognosis, we analyzed the correlation between these two biomarkers. Expressed BRAF V600E mutation correlated negatively with both SPINK1 mRNA expression level (Spearman's rho −0.19, *P* < 0.001) and the previously published (Koskensalo *et al*., 2012) IHC results (Spearman's rho −0.21, *P* < 0.001). The relative SPINK1 mRNA expression was lower in samples with the expression of mutated BRAF V600E (median 0.4, IQR 0.1–0.6) than in samples with BRAF wild‐type expression only (median 0.8, IQR 0.3–1.5, *P* = < 0.0001, Mann–Whitney test). The hazard ratio of high SPINK1 mRNA level (> 2.4) in patients with expression of wild‐type BRAF to patients with expressed BRAF V600E mutations was 0.09 (95% CI, 0.03–0.27) after adjusting for age, gender, stage, and location. All patients, except one, with expressed BRAF V600E mutations had low SPINK1 mRNA levels (≤ 2.4 relative expression).

### 3.4 Effect of MAPK inhibitors on SPINK1 secretion

Next, we analyzed the levels of secreted SPINK1 and its putative serine protease targets trypsin‐1 and trypsin‐2 in a panel of CRC and melanoma cell lines harboring either wild‐type or V600E BRAF. Table 4 shows the basal levels of SPINK1, trypsin‐1, and trypsin‐2 at 72‐h time point in a panel of cell lines and their respective BRAF status. Notably, neither of the melanoma cell lines secreted SPINK1 or trypsins.

**Table 4:** Basal levels of secreted SPINK1, trypsin‐1 and ‐2 at 72‐h time point

Cancer type	Cell line	BRAF status	SPINK1, ng·mL^−1^	Trypsin‐1, ng·mL^−1^	Trypsin‐2, ng·mL^−1^
CRC	Colo205	V600E	1.6 (± 0.06)	12.2 (± 0.8)	56 (± 6.2)
CRC	HT‐29	V600E	11.4 (± 2.4)	2.5 (± 0.3)	3.9 (± 0.5)
CRC	Caco‐2	WT	35.3 (± 4.3)	1.3 (± 0.3)	ND
CRC	SW480	WT	ND	ND	ND
Melanoma	SK‐MEL‐2	WT	ND	ND	ND
Melanoma	SK‐MEL‐5	V600E	ND	ND	ND

**Figure 3:**
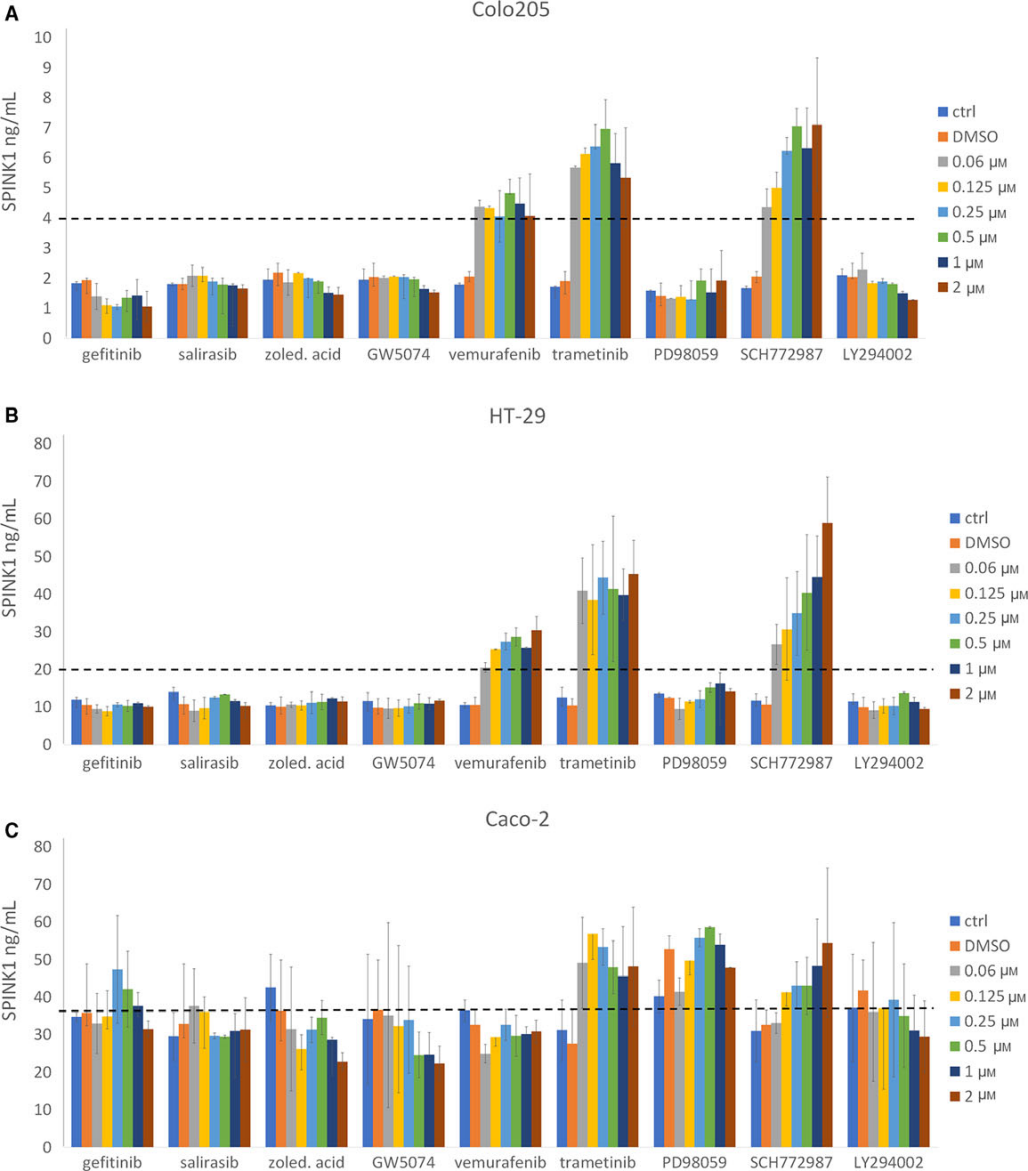
MAPK pathway inhibitors increase SPINK1 levels in BRAF V600E colorectal cancer. Secreted SPINK1 protein levels were analyzed by IFMA in Colo205 (A), HT‐29 (B), and (C) Caco‐2 cells at 72‐h time point. Vemurafenib, trametinib, and SCH772984 increased SPINK1 secretion in Colo205 and HT‐29 cells over twofold (dashed line), whereas no twofold increase was seen in the Caco‐2 cells compared to control and DMSO‐treated cells.

In order to study the effects of various MAPK pathway inhibitors on the SPINK1 levels, CRC cell lines were treated with the following compounds: gefitinib, GW5074, LY294002, PD98059, salisarib, SCH772984, trametinib, vemurafenib, and zoledronic acid. In BRAF V600E CRC cells lines Colo205 and HT‐29, inhibitors affecting the MAPK pathway at or below BRAF resulted in over twofold dose‐dependent increase in SPINK1 secretion measured at 72‐h time point (Fig. 3A,B). This effect was seen with the BRAF inhibitor vemurafenib and subsequent MAPK pathway inhibitors trametinib (MEK1/2 inhibitor) and SCH772984 (ERK1/2 inhibitor), but interestingly not with the CRAF inhibitor GW5074 or with PD98059 that is a non‐ATP competing MEK antagonist specifically inhibiting MEK1‐mediated activation of the MAPK pathway. Further, inhibitors upstream of RAF, such as the RAS inhibitor salisarib, RAS/RHO inhibitor zoledronic acid, or EGFR inhibitor gefitinib did not induce SPINK1 secretion in BRAF V600E CRC cells. These effects were not seen in the BRAF wild‐type CRC cell line Caco‐2 (Fig. 3C). Akt inhibitor LY294002 did not affect SPINK1 levels in any of the tested cell lines.

### 3.5 MAPK inhibitors induce SPINK1 and concomitantly downregulate trypsin‐1 and ‐2 in BRAF V600E cells

**Figure 4:**
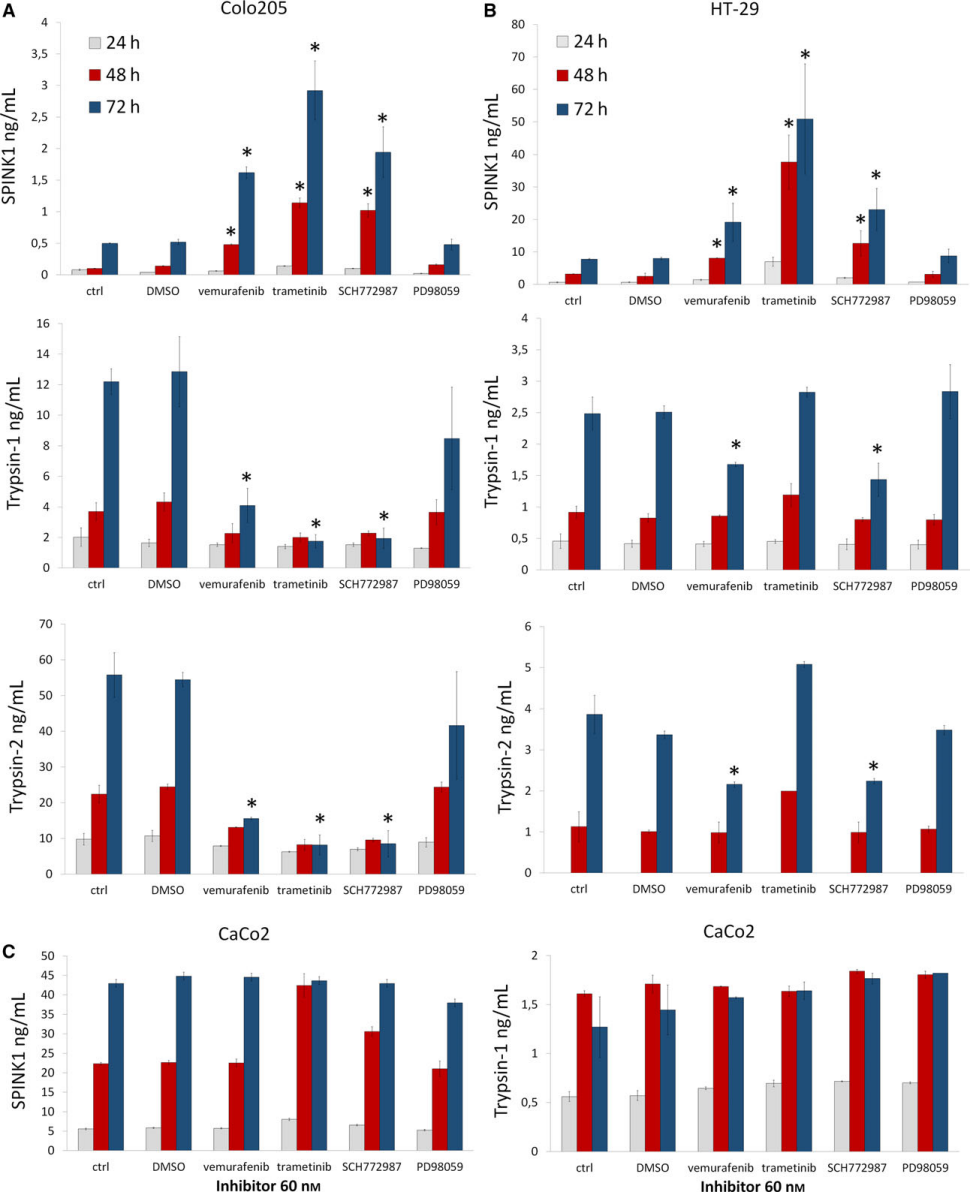
Time‐dependent increase in SPINK1 levels in response to vemurafenib, trametinib, and SCH772984 in Colo205 and HT‐29 cells. (A) In Colo205 cells, vemurafenib, trametinib, and SCH772984 significantly increased SPINK1 secretion at 48‐ and 72‐h time points with a concomitant decrease in trypsin‐1 and ‐2 secretion at 72‐h time point as measured by respective IFMAs. (B) In HT‐29 cells, vemurafenib, trametinib, and SCH772984 significantly increased SPINK1 secretion at 48‐ and 72‐h time points. Vemurafenib and SCH772984, but no trametinib, reduced trypsin‐1 and ‐2 levels in HT‐29 cells at 72‐h time point. (C) Vemurafenib, trametinib, and SCH772984 did not increase SPINK1 secretion or decrease trypsin‐1 secretion in Caco‐2 cells compared to control or DMSO‐treated cells. Significantly different (*P < 0.05) as compared to control by two‐tailed *t*‐test. Trypsin‐2 was not detected in HT‐29 cells at 24‐h time point and in Caco‐2 cells at any time point.

To further elucidate the effects of the MAPK pathway inhibitors on CRC cells, using the minimum dose that induced SPINK1 secretion in Colo205 in HT‐29 cells (60 nm), we measured the levels of SPINK1 in a time‐dependent manner. At 48‐ and 72‐h time points in both cell lines, vemurafenib, trametinib, and SCH772984 resulted in a statistically significant increase in SPINK1 secretion (*P* < 0.05) compared to control, as measured by IFMA (Fig. 4A,B, top panels). Corroborating the immunoassay results, SPINK1 mRNA levels were increased at the 72‐h time point in response to vemurafenib, trametinib, and SCH772984 in both cell lines (Fig. S1A).

Furthermore, as SPINK1 is a putative trypsin inhibitor, we investigated whether the MAPK inhibitors affected endogenous trypsin levels in the CRC cell lines. In the BRAF V600E Colo205 cells, vemurafenib, trametinib, and SCH772984 led to a statistically significant (*P* < 0.05) decrease in trypsin‐1 (Fig. 4A, mid‐panel) and trypsin‐2 (Fig. 4A, bottom panel) levels at 72‐h time point, suggesting inverse regulation between SPINK1 and its target proteases. In the other BRAF V600E cell line HT‐29 (Fig. 4B), vemurafenib and SCH772984 led to statistically significant (*P* < 0.05) decrease in trypsin‐1 and ‐2 levels, whereas in trametinib‐treated cells trypsin‐1 and ‐2 levels did not decrease. These data were confirmed by qPCR analyses of the mRNA levels of PRSS1 (trypsin‐1) and PRSS2 (trypsin‐2) (Fig. S1B, C).

In the BRAF wild‐type cell line Caco‐2, level of SPINK1 was slightly increased in response to trametinib at 48‐h time point, but not in response to vemurafenib and SCH772984, as analyzed by IFMA (Fig. 4C). Trypsin‐1 levels were not affected by inhibitors (Fig. 4C, right panel) and trypsin‐2 was not detected in the Caco‐2 cell conditioned media by IFMA. The results were confirmed by qPCR analyses of the mRNA levels of SPINK1, PRSS1 (trypsin‐1), and PRSS2 (trypsin‐2), indicating a correlation between mRNA expression and secretion of these proteins (Fig. S1).

### 3.6 Trametinib diminishes ERK1/2 and MEK1/2 phosphorylation and downregulates ATF‐4

**Figure 5:**
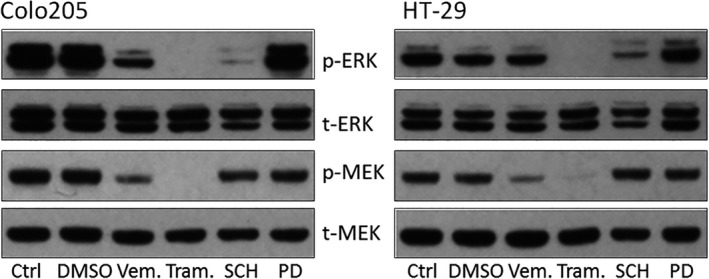
Trametinib diminishes ERK and MEK phosphorylation in BRAF V600E CRC cells. ERK1/2 (Thr202/Tyr204) and MEK1/2 (Ser217/221) residues are dephosphorylated by trametinib (60 nm) as shown by a western blot of whole‐cell lysates of Colo205 and HT‐29 cell lines at 24‐h time point. Vemurafenib and SCH772984 reduce ERK1/2 (Thr202/Tyr204) and MEK1/2 (Ser217/221) phosphorylation to a lesser extent in Colo205 and HT‐29 cells. Vemurafenib (Vem.), trametinib (Tram.), SCH772984 (SCH), or PD98059 (PD). Total ERK1/2 and MEK1/2 antibodies were used as controls.

To elucidate the signaling events that led to increased SPINK1 expression in response to MAPK inhibitors, we studied the phosphorylation status of MEK1/2 and ERK1/2 at 24‐h time point (Fig. 5). In both Colo205 and HT‐29 cells, trametinib diminished completely the phosphorylated forms of ERK1/2 (Thr202/Tyr204) and MEK1/2 (Ser217/221). However, vemurafenib and SCH772984 reduced phosphorylation of ERK1/2 and MEK1/2 to a much lesser extent compared to trametinib. These results were in line with the observed increase in SPINK1 levels in these cells. Further, PD98059, which did not affect SPINK1 expression (Fig. 4), did not affect the phosphorylation status of ERK1/2 or MEK1/2 in either CRC cell line.

**Figure 6:**
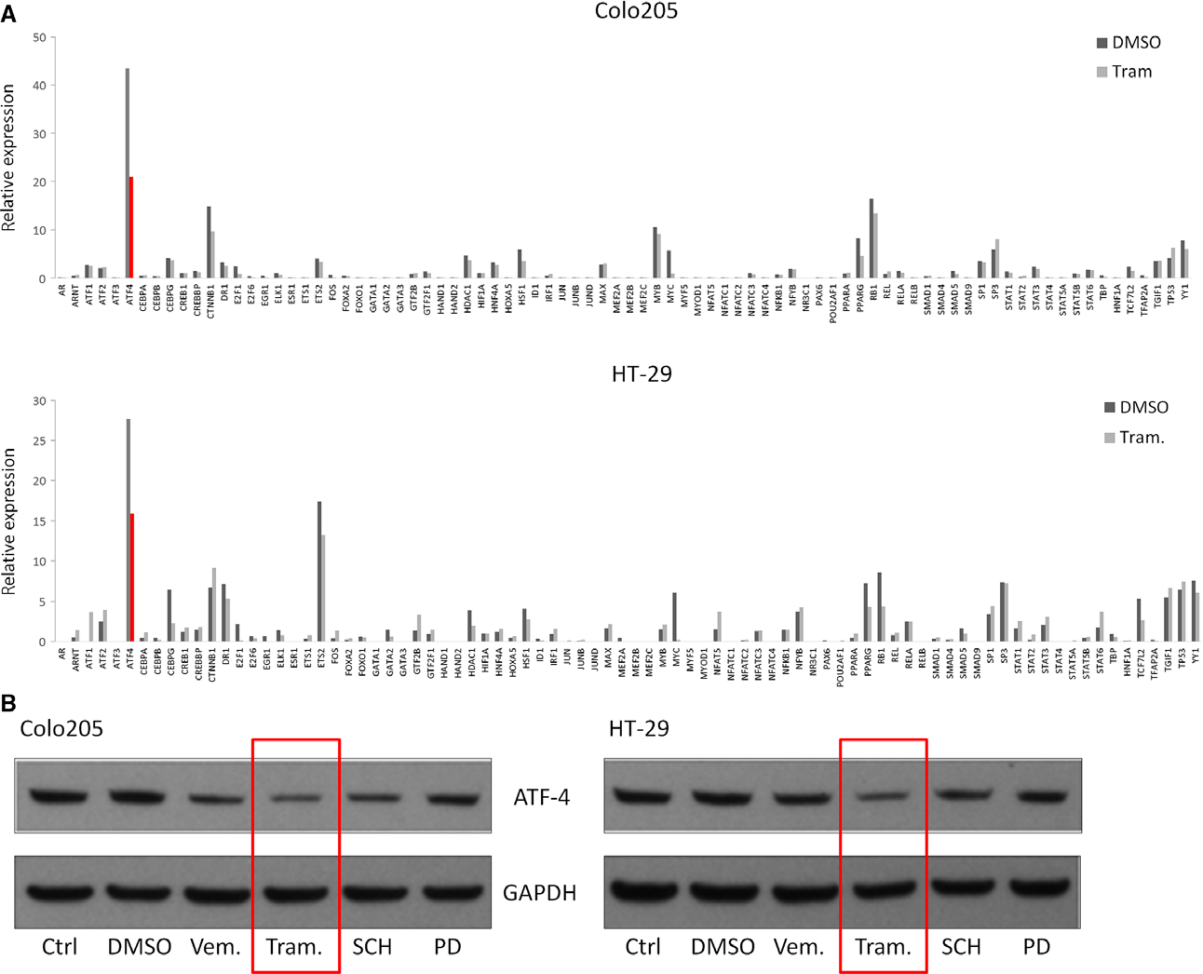
Trametinib downregulates ATF‐4 mRNA and protein levels. (A) Colo205 and HT‐29 cells were treated with 60 nm trametinib for 24 h after which RNA was extracted and TaqMan non‐HOX transcription factor array was performed. ATF‐4 mRNA was decreased by twofold in response to trametinib when compared to DMSO control. (B) Western blot of whole‐cell lysates of Colo205 and HT‐29 cells harvested after 24‐h treatment with either 60 nm vemurafenib (Vem.), trametinib (Tram.), SCH772984 (SCH), or PD98059 (PD) showing downregulation of ATF‐4 protein level in response to vemurafenib. GAPDH was used as a loading control.

Next, we performed a TaqMan non‐HOX transcription factor array in order to identify which transcription factor is responsible for the increased SPINK1 expression. As trametinib caused the biggest increase in SPINK1 level in both cell lines, we used it at 60 nm concentration and harvested RNA at 24‐h time point. Interestingly, none of the transcription factors included in the array were significantly induced in the trametinib‐treated Colo205 and HT‐29 cells (Fig. 6A). The only transcription factor with a change in its mRNA level in the trametinib‐treated sample compared to the DMSO control was activating transcription factor 4 (ATF‐4), a transcription factor linked to integrated stress response (ISR) (Pakos‐Zebrucka *et al*., 2016). The decrease in ATF‐4 caused by trametinib was further confirmed by western blotting (Fig. 6B).

As we have previously shown that interleukin‐6 induces SPINK1 expression in Colo205 and HT‐29 cells via STAT3 phosphorylation (Räsänen *et al*., 2016b), we investigated whether this was also the case with the MAPK inhibitors. Phosphorylation of STAT3 was not induced in response to the inhibitors (data not shown) and the total STAT3 levels remained constant (Fig. S2).

## 4 Discussion

CRC patients with the BRAF V600E mutation present a clinical challenge, as no effective treatments have been found for this subpopulation. In keeping with previously published results (Barras, 2015), patients with this mutation have a decreased survival rate in our cohort. Several publications have shown that SPINK1 plays a role in the tumorigenesis of CRC, in particular at the later stages (Räsänen *et al*., 2016a). In this study, we demonstrate that SPINK1 protein and mRNA levels correlate and that low SPINK1 expression in tumor tissue is indicative of poor prognosis, in line with our previously published results (Koskensalo *et al*., 2012) and with a recent publication (Chen *et al*., 2016) in which high SPINK1 tumor expression correlated with a good prognosis in patients with CRC receiving cetuximab therapy. Further, here we show for the first time that expression of BRAF V600E mutation correlates with low SPINK1 expression level. The ExBP‐RT method used for the BRAF V600E analyses detects mRNA of expressed mutations in tumor tissue, rather than the presence of mutated DNA. This allowed us to use the same patient samples to analyze the expression of both SPINK1 and BRAF V600E mRNA.

These clinical findings of BRAF V600E and SPINK1 expression in our CRC patient cohort led us to hypothesize that MAPK inhibitors might affect SPINK1 levels. In order to test this, we used a panel of CRC cell lines harboring the V600E‐mutant BRAF and compared the effects to a BRAF wild‐type CRC cell line. Vemurafenib treatment is not beneficial in BRAF‐mutant CRC patients and combination therapies with MAPK inhibitors with EGFR inhibitors are under clinical investigations. A phase I study by Corcoran *et al*. (2015) suggested that dual MAPK pathway blockade with the BRAF inhibitor dabrafenib and the MEK inhibitor trametinib can lead to a meaningful clinical benefit in a subset of patients with BRAF V600E metastatic CRC. Based on our results, trametinib treatment, which inhibits both MEK1 and MEK2, might be an effective therapy in BRAF V600E‐positive/SPINK1‐low subpopulation of patients with CRC, as it led to increased SPINK1 secretion in BRAF V600E‐positive CRC cells. Furthermore, it was the only MAPK inhibitor that was able to diminish phosphorylation of MEK and ERK in the BRAF V600E CRC cell lines Colo205 and HT‐29. Our data also support both preclinical and clinical findings that vemurafenib is not effective in BRAF‐mutant CRC, as it was not capable to completely suppress MAPK signaling. As PD98059, a MEK1 inhibitor, was not able to affect SPINK1 secretion or MEK and ERK phosphorylation, our data implicate MEK2 as a critical protein in the MAPK pathway in colorectal adenocarcinoma.

Bidirectional kinase–protease interactions are known to have a role in cancer and clinical implications of such kinase–protease crosstalk have started to emerge (Lopez‐Otin and Hunter, 2010). In our study, parallel to SPINK1 increase, we observed a decrease in the expression and secretion of trypsin‐1 and ‐2 in response to vemurafenib, trametinib, and SCH772984. Studies have suggested that robust MAPK pathway suppression is required for response in BRAF V600E cancers and acquired resistance to BRAF inhibitor combinations involve reactivation of the MAPK pathway (Ahronian *et al*., 2015). Recently, Miller *et al*. (2016) showed that MEK inhibitors lead to a reduced proteolytic shedding of cell surface receptor tyrosine kinases by inhibiting the catalytic activity of a disintegrin and metalloproteinases (ADAM), thus leading to increased mitogenic signaling and kinase inhibitor resistance. Further, disrupting the protease inhibition by neutralizing a putative ADAM10 inhibitor tissue inhibitor of metalloproteinases 1 (TIMP1), MAPK inhibitor efficacy was improved (Miller *et al*., 2016). These findings, along with ours, highlight the extensive crosstalk between kinases, proteases, and cognate protease inhibitors in response to molecularly targeted therapies and warrant further studies.

Here, we describe a novel mechanism of ATF‐4 transcription repression by molecularly targeted therapy, as trametinib was able to downregulate ATF‐4 transcription leading to reduced ATF‐4 protein level in Colo205 and HT‐29 cells. ATF‐4 is a well‐characterized effector of ISR. It has several dimerization partners that influence its gene transcription, thus guiding cellular outcomes (Pakos‐Zebrucka *et al*., 2016). In most cases, cellular stress induces upregulation of ATF‐4 transcription. For example, in BRAF inhibitor‐sensitive melanoma cell lines, the preclinical version of vemurafenib, PLX4720, led to a rapid induction of ATF‐4 (Ma *et al*., 2014). However, there is evidence of transcriptional repression of ATF‐4 by some cellular stressors, such as C/EBPβ during UV irradiation and in nonalcoholic fatty liver and nonalcoholic steatohepatatis (Pakos‐Zebrucka *et al*., 2016). Of note, the mechanism by which these MAPK inhibitors activate SPINK1 transcription remains to be revealed, as on the transcription factor array we did not observe any significant increases in response to the trametinib treatment.

## 5 Conclusions

In conclusion, this study demonstrates for the first time an inverse relationship between expressed BRAF V600E mutations and SPINK1 expression. Further, we show that in addition to downregulating phosphorylation of ERK and MEK, trametinib treatment leads also to downregulation of ATF‐4 and trypsin‐1 and ‐2 with a concomitant increase in SPINK1 secretion. Both ATF‐4 and trypsins have been shown to confer survival advantage of cancer cells and thereby to regulate tumor progression. Thus, finding an effective way to inhibit the expression of these proteins while sustaining SPINK1 levels might have a clinical benefit in BRAF V600E‐positive colorectal adenocarcinoma. Although further studies are warranted, SPINK1 expression seems to be a useful biomarker in CRC and its expression might guide patient stratification and treatment response to molecularly targeted therapies.

## Author contributions

KR and JS were responsible for the study conception, design, and data analysis. SL and CH were responsible for the patient identification and sample collection. KXD and THH were responsible for the ExBP‐RT data acquisition. KR was responsible for the *in vitro* studies. HM was responsible for the statistical analyses. All authors were responsible for the data interpretation and manuscript writing. All authors read and approved the final version of the manuscript.

## Supporting information


**Fig. S1.** (A) *SPINK1*, (B) *PRSS1* and (C) *PRSS2* mRNA levels analyzed by qPCR in response to inhibitor treatment (60 nm) at 72 h time point.

**Fig. S2.** Western blot of whole‐cell lysates of Colo205 and HT‐29 cells harvested after 24 h treatment with either 60 nm vemurafenib (Vem.), trametinib (Tram.), SCH772984 (SCH) or PD98059 (PD).
