# Supplementary figures and images for: MAPK inhibitors induce serine peptidase inhibitor Kazal type 1 (SPINK1) secretion in BRAF V600E‐mutant colorectal adenocarcinoma
Source: Mol Oncol. 2017 Dec 27;12(2):224–38. doi: 10.1002/1878-0261.12160 (PMC5792734; doi:10.1002/1878-0261.12160)

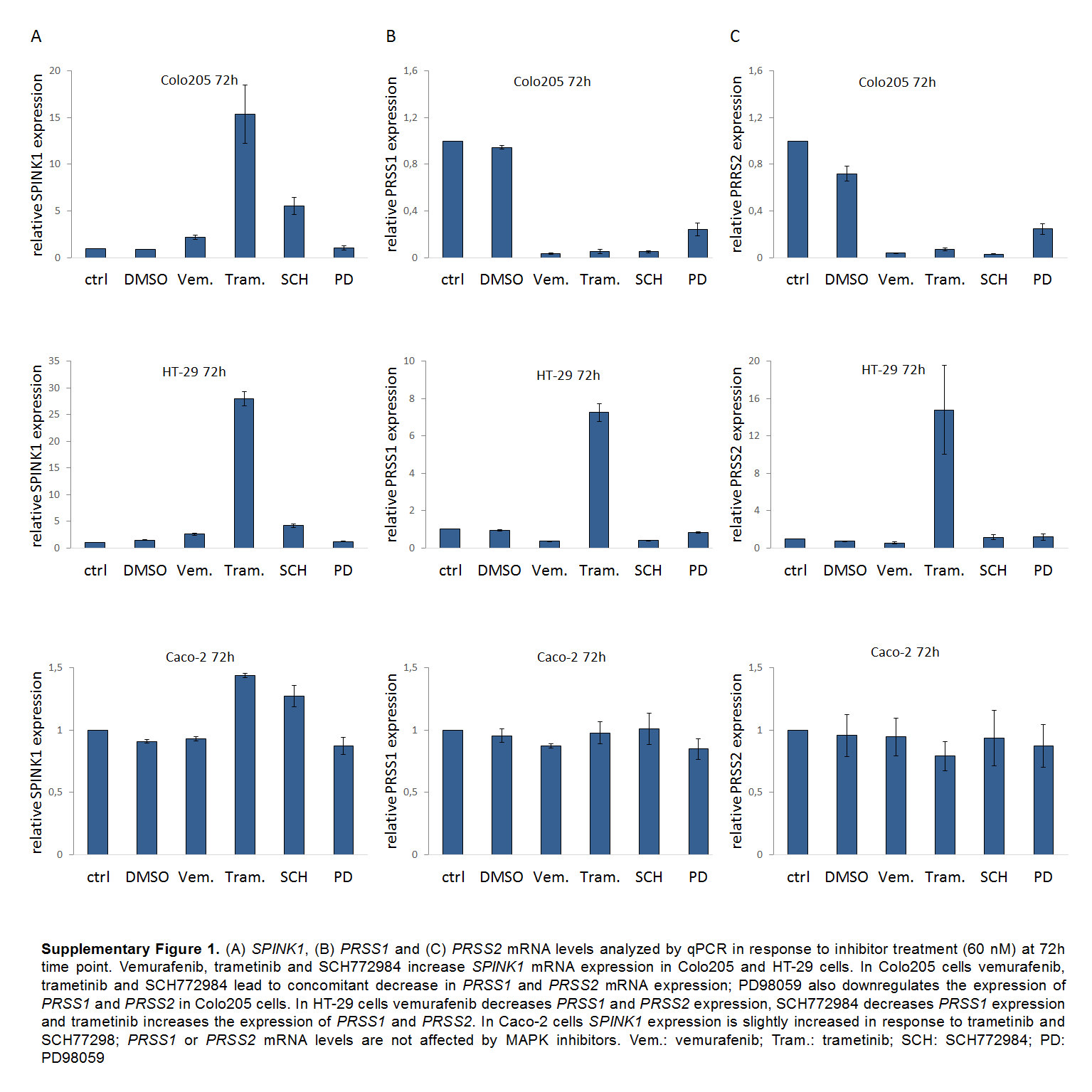

Supplement: Supplementary file 1 — Fig. S1. (A) SPINK1, (B) PRSS1 and (C) PRSS2 mRNA levels analyzed by qPCR in response to inhibitor treatment (60 nm) at 72 h time point. [file MOL2-12-224-s001.tif]

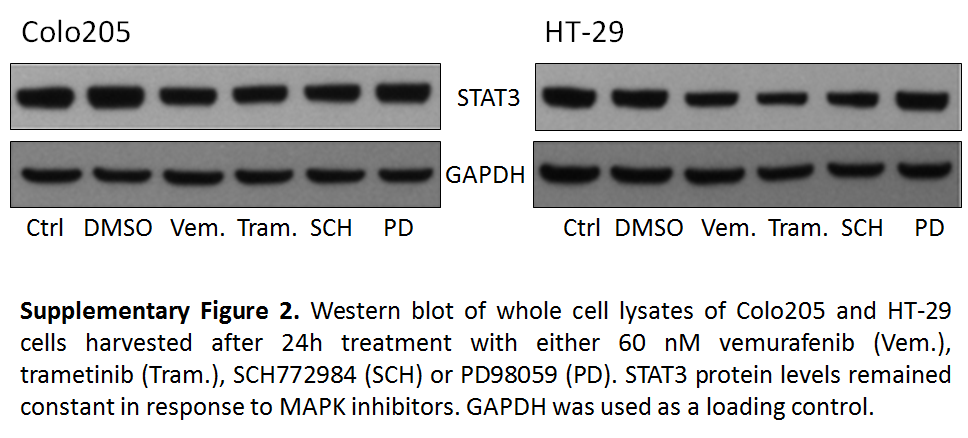

Supplement: Supplementary file 2 — Fig. S2. Western blot of whole‐cell lysates of Colo205 and HT‐29 cells harvested after 24 h treatment with either 60 nm vemurafenib (Vem.), trametinib (Tram.), SCH772984 (SCH) or PD98059 (PD). [file MOL2-12-224-s002.tif]
